# EXAMINING A PERSON-CENTERED MODEL OF COMMUNITY SUPERVISION FOR OLDER ADULTS

**DOI:** 10.1093/geroni/igad104.1476

**Published:** 2023-12-21

**Authors:** Victoria Helmly

**Affiliations:** Georgia State University, Atlanta, Georgia, United States

## Abstract

Though research in aging and the criminal legal system is growing, there is still limited data on how the carceral system impacts this population. In this presentation, I will discuss my dissertation research on older adults on parole and probation (community supervision). This qualitative research study examines both the experiences of older adults under community supervision and the experiences of the officers who supervise this population. This data is collected through semi-structured interviews with community supervision officers and older adults under supervision. In addition to learning about the unique experiences of older adults impacted by the criminal legal system, this research aims to better understand a “person-centered model of community supervision” in the Southeastern United States. The interviews inquire about how this model is implemented, perceived, and understood by the participants. This research aims to inform policy and practice of community supervision and stimulate additional research in this area. In this presentation, I will discuss the study’s relevance to Gerontology, the research methodology, and the findings from the participant interviews. I will also discuss how this research can inform practice and future studies.

